# The pregnancy outcomes of infertile women with polycystic ovary syndrome undergoing intrauterine insemination with different attempts of previous ovulation induction

**DOI:** 10.3389/fendo.2022.922605

**Published:** 2022-08-22

**Authors:** Yining Gao, Shutian Jiang, Li Chen, Qianwen Xi, Wenzhi Li, Shaozhen Zhang, Yanping Kuang

**Affiliations:** Department of Assisted Reproduction, Shanghai Ninth People’s Hospital, Shanghai Jiao Tong University School of Medicine, Shanghai, China

**Keywords:** polycystic ovary syndrome (PCOS), intrauterine insemination (IUI), pregnancy outcomes, ovulation induction, predictive factor

## Abstract

**Background:**

Polycystic ovary syndrome (PCOS) is one of the most common reasons for infertility. The consensus of the treatment of infertile women with PCOS is ovulation induction (OI) for six to nine attempts before *in vitro* fertilization (IVF)/intracytoplasmic sperm injection (ICSI). Nowadays, more attention was paid to a rising, noninvasive treatment, intrauterine insemination (IUI), as some experts claimed IUI could benefit PCOS patients with infertility. Our study means to investigate the outcomes of IUI for PCOS patients and if patients’ previous OI cycles can be a predictive factor for IUI outcomes.

**Methods:**

A total of 1,086 PCOS patients was included and 1,868 IUI cycles were performed between January 2007 and July 2021 in the department of assisted reproduction in Shanghai Ninth People’s Hospital. All included patients underwent IUI treatments with letrozole+human menopausal gonadotropin (LE+hMG) for ovarian stimulation.

**Results:**

The pregnancy outcomes were not associated with the attempts of failed OI cycles previously. Specifically, the clinical pregnancy rate was 21.14% for PCOS patients without previous OI cycles, 21.95% for PCOS patients with 1-2 previous OI cycles and 23.64% for PCOS patients with 3 or more previous OI cycles (p=0.507). The corresponding live birth rate was 16.64%, 18.06%, and 18.68%, respectively, of which the difference was not statistically significant (p=0.627). The cumulative rate per patient was 38.59% for clinical pregnancy and 31.03% for live birth, and approximately 98% of the pregnancies occurred in the first 3 cycles of IUI.

**Conclusion:**

PCOS women with different attempts of OI cycles had similar pregnancy outcomes after IUI, thus a history of repeated failures of OI treatments was not a predictive factor for the pregnancy outcomes in IUI cycles. Most pregnancies occurred in the first three cycles of IUI, so we strongly recommended three attempts of IUI for PCOS women before they switched to IVF/ICSI. Generally, IUI might be an assist for infertile women with PCOS before IVF/ICSI and might accelerate pregnancy for target women without invasive manipulations.

## Introduction

Polycystic ovary syndrome (PCOS) is one of the most common reasons for infertility worldwide that affects 8%–19% of reproductive-aged women and accounts for ~80% of anovulatory infertility (1). PCOS is a systematic syndrome with metabolic, psychologic, and reproductive features. According to the 2003 Rotterdam Criteria, PCOS is diagnosed with two of three features: oligo- or anovulation, polycystic ovarian morphology on ultrasound, and hyperandrogenism (clinical or biochemical). Other clinical features include obesity, insulin resistance (IR), type II diabetes (DM2) (2), abnormal menstrual cycles, and acne.

WHO proposed a global guidance on the management of infertile women with PCOS in 2016, which recommended taking oral ovulation induction (OI) agents as the first-line therapy, such as Clomiphene Citrate (CC), for 6-9 cycles of attempts (3, 4). If patients detected no ovulation through this procedure, gonadotropin could be used as a second-line therapy, as well as letrozole (LE) and CC+metformin (3). In 2018, Australian National Health and Medical Research Council (NHMRC) developed another evidence-based guideline for infertile women with PCOS. It suggested that LE should be considered as first-line therapy, for improving pregnancy rates and avoiding adverse effects (1). Artificial reproductive techniques (ART), including *in vitro* fertilization (IVF) and intracytoplasmic sperm injection (ICSI), were required for women with PCOS who resisted OI therapy or failed to get pregnant after 6-9 cycles of OI therapy. Although with its high pregnancy rate, IVF/ICSI also displays weakness of high cost, complex procedure, as well as higher risk of ovarian hyper-stimulation syndrome (OHSS) (5). Additionally, even if IVF/ICSI is to be adopted, the indication and timing of clinical practice are still under debate. Some experts recommend six to nine attempts of OI therapy, whereas some Chinese experts suggest no more than six attempts before IVF/ICSI (6).

In recent years, some randomized controlled trials focused on a less invasive ART, intrauterine insemination (IUI), for infertile women with PCOS. A study showed that the biochemical and clinical pregnancy rates of PCOS patients undergoing IUI after OI by CC were significantly higher than patients undergoing timed-intercourse (TIC) after CC (7). This result indicated that IUI might increase the pregnancy potential of PCOS women, whereas the relevant studies were limited. Other studies for PCOS patients undergoing IUI focused more on the factors that will affect the pregnancy outcomes of IUI treatment. For instance, it was shown that different OI drugs, including LE, CC, and human menopausal gonadotropin (hMG), did not affect the pregnancy rate of IUI for PCOS patients (8). Besides, the diameters of leading follicles were not related to the pregnancy outcomes of PCOS patients undergoing IUI once the leading follicles reached 17 mm in diameters (9), whereas the endometrial thicknesses had a significantly positive correlation with the pregnancy outcomes for PCOS in IUI (10). Despite a variety of previous work on PCOS women undergoing IUI, there was no evidence on IUI outcomes of PCOS women with a history of repeated OI cycles. As repeated OI failures may be considered as an indication for IVF/ICSI, suggesting the subfertility of this subgroup of PCOS patients, it is necessary to figure out whether PCOS patients’ previous OI attempts can also be a predictive factor for pregnancy outcomes in IUI. Yet, research are very limited in this area.

Therefore, this single-centered retrospective study means to investigate whether IUI can be an effective treatment for infertile patients with PCOS and if the previous OI cycles can be a predictive factor for the pregnancy outcomes of infertile women with PCOS undergoing IUI. Furthermore, we aim to explore how many attempts of IUI are appropriate before a PCOS patient switches to IVF/ICSI.

## Material and methods

### Patients

According to Rotterdam Criteria (2003), patients met the following criteria (two out of three) were diagnosed as PCOS in our study: 1) oligo- and/or anovulation, 2) clinical and/or biochemical sign of hyperandrogenism, and 3) polycystic ovaries. Meanwhile, the diagnosis was made after the exclusion of other diseases, such as congenital adrenal hyperplasia and Cushing’s syndrome, etc. Between January 2007 and July 2021, all the PCOS patients with their age under 40 years old were enrolled in the study, involving a total of 1,086 PCOS patients and 1,868 IUI cycles in the department of assisted reproduction in Shanghai Ninth People’s Hospital. Bilateral oviduct obstruction or severe oligospermia, asthenospermia, and teratozoospermia were considered as contraindications for IUI, therefore patients with these causes of infertility were excluded. In addition, 3,222 cycles of IVF/ICSI for PCOS patients were included, based on the results of Propensity Score Matching (PSM). This study was approved by the Ethics Committee (Institutional Review Board) of Shanghai Ninth People’s Hospital.

Grouping of patients was based on the patients’ failed OI cycles previously. We classified our candidates into three groups: patients with no previous OI cycles, patients with 1-2 previous OI cycles, and patients with 3 or more OI cycles. Each patient’s basic characteristics were evaluated by Body Mass Index (BMI), duration of infertility, and hormone level on the third day of menstrual cycle. Cycle characteristics were measured by cycle length, endometrial thickness, hormone level, and follicle numbers on human chorionic gonadotropin (hCG) day.

### Sperm quality and preparation

Fresh semen from husbands was collected through masturbation into sterile containers after 3-7 days of abstinence. After 10-15 min of liquefaction at room temperature, the sample was washed with 3-layer density gradient centrifugation using Isolate (Irvine Scientific, USA). Sperm samples with a total sperm concentration of >10 million sperm/ml, a total post-wash sperm count >2 million. and a normal morphology ≥4% were considered qualified. After semen analysis, the final sample was suspended in a 0.5 ml medium and incubated for another 30-60 min. All samples were analyzed and prepared in the hospital laboratory.

### Ovulation induction

All cycles in our study underwent ovarian stimulation with the combination of LE and hMG. Ovulation induction started on the 3^rd^ day of menstruation with an initial dose of 2.5-5 mg of LE orally for 3-5 days and a further consultation on day 10 was needed. Once the leading follicle reached 10 mm in diameter, intramuscular injection of 75 IU of hMG was performed until the leading follicle reached a diameter of 18-20 mm. Ultrasound examinations were performed every 2-3 days to monitor the growth of follicles and hormone tests were also given at the same time. When a follicle’s diameter hit 18 mm and the number of dominate follicles was less than three, a dose of 5,000 IU human chorionic gonadotropin (hCG) was given to trigger ovulation.

### Insemination

Around 38 h after the administration of hCG, a single IUI was performed by an experienced clinical doctor. The patients were required to be in the lithotomy position during the operation. After regular disinfecting of the vulva and exposing the cervical, 0.5 ml of semen suspension was injected into the uterine cavity through a disposable injection syringe. The patients were recommended to stay in the supine position for 30 min after the operation.

### Pregnancy outcome evaluation

All patients were asked to take the urine hCG test 14 days after the insemination and come to the hospital for a review. A more accurate serum hCG test and an ultrasound examination would be given to confirm if the patients were pregnant. All pregnant patients would have follow-up visits through phone interviews until their delivery or abortion. Detailed birth outcomes and obstetric complications were recorded in all cases. Of note, since our follow-up ended in October 2021, only the neonatal outcomes in IUI cycles performed before December 2020 were followed up. Therefore, when calculating the live birth rate, only cycles completed before December 2020 were counted. For the IUI cycles performed in 2021, the follow-up continued to ongoing pregnancy.

### Statistical analysis

Data were expressed as mean ± standard deviation if it was in a normal distribution and were expressed as mean (quartile) if it was not normally distributed. All statistical analysis was performed using IBM SPSS 23. Student’s *t* test or one-way ANOVA was used for continuous variables as appropriate and chi-square test was used for categorical variables. Logistic regression was performed by setting the clinical pregnancy as the outcome variable. Results were considered significant at a P value <0.05. In addition, propensity score matching (PSM) was performed between PCOS patients undergoing IVF/ICSI and those undergoing IUI based on the estimated propensity scores using nearest-neighbor matching (1:2) within a caliper width of 0.1 standard deviation without replacement.

## Results

### Basic and cycle characteristics

Totally, 1,868 IUI cycles of 1,086 couples were included in this study, of which 667 cycles of 387 couples did not have previous OI cycle, 410 cycles of 247 couples had 1-2 failed OI cycles and 791 cycles of 456 couples had 3 or more failed OI cycles previously. The indication of those patients undergoing IUI was shown in **Table 1**. Around 30% of them had a combined causes of infertility (PCOS + male factors and PCOS + endometriosis), whereas the other 70% only suffered from PCOS, who might have undergone a long time of OI therapy and had a strong desire for IUI.

**Table 1:** Indications of PCOS patients undergoing IUI.

	No previous OI cycles (cycles/patients)	1-2 Previous OI cycles (cycles/patients)	≥3 previous OI cycles (cycles/patients)	P-value
PCOS	458/263	301/180	602/343	0.77
PCOS + Male Factors	198/114	102/63	211/130	
PCOS + Endometriosis	11/6	7/4	17/10	
Total	667/383	410/247	791/456	

Patients’ basic and cycle characteristics were presented in **Table 2**. The BMI of patients with ≥3 previous OI cycles was significantly less than the other two groups (24.79 ± 4.07 vs. 24.26 ± 3.96vs. 23.63 ± 3.80 kg/m^2^, p=0.007). Patients with ≥3 previous OI cycles had the longest duration of infertility. The average duration was 3.13 years, compared to 2.81 years and 2.76 years for patients with no previous OI cycles and 1-2 previous OI cycles, respectively.

**Table 2:** Comparison of baseline and cycle characteristics.

Variables		No previous cycle	1-2 previous cycles	≥3 previous cycles	P-Value
Patients/Cycles		383/667	247/410	456/791	/
Age (years)		33.09 ± 3.79	33.31 ± 3.55	33.47 ± 3.27	0.164
BMI (kg/m^2^)		24.79 ± 4.07	24.26 ± 3.96	23.63 ± 3.80	0.007*
Duration of Infertility (years)		2.81 (1)	2.76 (1)	3.13 (2)	0.005*
Basal Hormone Level	FSH (IU/L)	5.33 ± 1.29	5.18 ± 1.25	5.22 ± 1.27	0.189
	LH (IU/L)	5.09 (2.89)	5.23 (3.14)	5.50 (3.19)	0.063
	E2 (pg/ml)	32.19 (23)	34.36 (24)	33.91 (23)	0.073
	P (ng/ml)	0.25 (0.2)	0.24 (0.2)	0.26 (0.2)	0.17
Cycle Length (days)		12.90 ± 3.55	12.96 ± 3.43	12.55 ± 3.46	0.128
Endometrial Thickness on hCG day (mm)		10.03 ± 2.28	9.68 ± 2.26	9.88 ± 2.20	0.068
No. of Follicles >10 mm (n.)		2.87 (1)	2.77 (1)	2.65 (1)	0.314
No. of Follicles >14 mm (n.)		1.62 (1)	1.66 (1)	1.51 (1)	0.068
No. of Follicles >18 mm (n.)		0.67 (0)	0.64 (0)	0.68 (0)	0.380
Hormone Level on hCG day	FSH (IU/L)	7.13 ± 2.74	7.04 ± 2.52	6.93 ± 2.40	0.455
	LH (IU/L)	12.58 (5.775)	12.68 (5.68)	11.79 (5.85)	0.323
	E2 (pg/ml)	322.96 (152)	332.31 (147.5)	295.74 (158)	0.382
	P (ng/ml)	0.48 (0.2)	0.51 (0.2)	0.49 (0.2)	0.178
Average E2 level (pg/ml)		198.21 (119)	231.39 (113)	211.26 (121)	0.860

All continuous variables were presented as mean ± SD; Others were presented as mean (quartile). Average E2 level = E2 level on hCG day/No. of follicles >14mm; *; significant differences.

Other characteristics were all comparable among the three groups, including the maternal age, cycle length, and endometrial thickness, follicle numbers, basal hormone levels and hormone levels on hCG days.

### Pregnancy outcomes

The pregnancy outcomes among different groups did not have a significant difference. As is displayed in **Table 3**, the biochemical pregnancy rate per cycle was 24.29%/25.37%/25.28% for PCOS patients with no/1-2/3 or more attempts of previous OI cycles, respectively (p=0.886). The clinical pregnancy rate followed a similar pattern, 21.14% (141/667) for PCOS patients with no previous OI cycles, 21.95%(90/410) for PCOS patients with 1-2 previous OI cycles and 23.64%(187/791) for PCOS patients with 3 or more previous OI cycles (p=0.507). The ongoing pregnancy rate was also comparable among the three groups (p=0.626). The live birth rate per cycle was 16.64% (95/571)/18.06% (65/360)/18.68% (139/744) for PCOS patients with no/1-2/3 or more previous OI cycles (p=0.627).

**Table 3:** Pregnancy outcomes of PCOS patients with different attempts of OI.

Outcomes	No previous cycle	1-2 previous cycles	≥3 previous cycles	P-Value
No. (%) of Biochemical Pregnancy	162/667 (24.29%)	104/410 (25.37%)	200/791 (25.28%)	0.886
No. (%) of Clinical Pregnancy	141/667 (21.14%)	90/410 (21.95%)	187/791 (23.64%)	0.507
No. (%) of Ongoing Pregnancy	124/667 (18.59%)	80/410 (19.51%)	163/791 (20.61%)	0.626
No. (%) of Ectopic Pregnancy	4/667 (0.60%)	6/410 (1.46%)	2/791 (0.25%)	0.044*
No. (%) of SAB	18/141 (12.77%)	13/90 (14.44%)	24/187 (12.83%)	0.920
No. (%) of Multiple Pregnancy	23/141 (16.31%)	12/90 (13.33%)	17/187 (9.09%)	0.140
No. (%) of Live Birth	95/571 (16.64%)	65/360 (18.06%)	139/744 (18.68%)	0.627

SAB, spontaneous abortion; Ongoing pregnancy; pregnancy lasts longer than 12 weeks; *; significant differences.

The multiple pregnancy rates and spontaneous abortion (SAB) rates among the three groups was not significantly different (p=0.140, p=0.920, respectively). As for the ectopic pregnancy, significant difference was detected. In detail, the ectopic pregnancy rate was the highest in PCOS patients with 1-2 previous OI cycles, which was 6 out of 410 cycles, and was 4/667 for PCOS patients with no previous OI cycles, and 2/791 for PCOS patients with more than 3 previous OI cycles. (1.46% vs 0.6% vs 0.25%, p=0.044).

**Figure 1:**
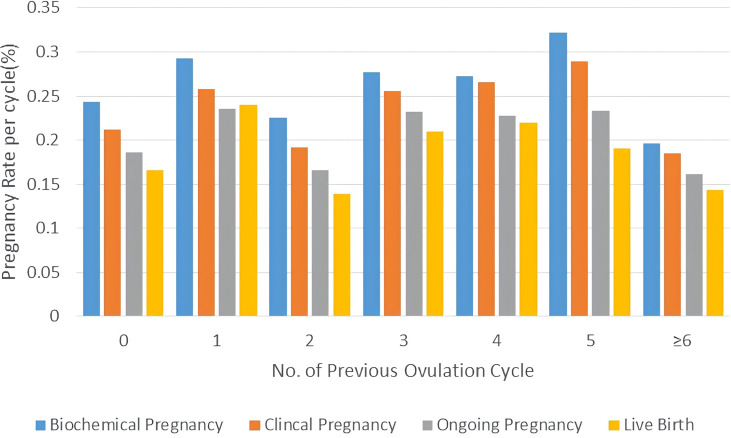
Pregnancy outcomes of PCOS patients with different attempts of OI in column charts. Pregnancy outcomes were as follows: biochemical pregnancy, clinical pregnancy, ongoing pregnancy, and live birth.

Logistic regression analysis was performed to assess the confounding factors that might have an influence on the clinical pregnancy, as detailed in **Table 4**. The results showed that PCOS patients’ previous OI cycles did not have a significant effect on clinical pregnancy rate with p-values at 0.864, 0.664, and 0.895. Patients’ No. of Follicles >14mm on hCG day also had a notable positive effect on clinical pregnancy rate after IUI with a p-value at 0.042 (OR=1.156). Moreover, patients’ endometrial thickness on hCG day had a remarkable positive impact on clinical pregnancy rate of IUI for PCOS women with a p-value at 0.009 (OR=1.093). A column chart was displayed to illustrate that there was no significant correlation between previous OI cycles and pregnancy outcomes of IUI for PCOS patients including biochemical/clinical/ongoing pregnancy and live birth. (**Figure 1**)

**Table 4:** Logistic regression analysis for pregnancy outcomes of PCOS patients undergoing IUI.

		B	Se	P	OR	CI (95%)	
						lower	upper
Previous OI cycles (n.)							
No previous OI cycle				.864			
1-2 previous OI cycles		.075	.172	.664	1.078	.769	1.510
≥3 previous OI cycles		-.024	.184	.895	.976	.680	1.401
Age(years)		-.033	.021	.119	.968	.929	1.008
Duration of infertility(years)		-.032	.036	.374	.969	.903	1.039
BMI(Kg/m2)		.013	.011	.219	1.013	.992	1.035
Basal Hormone Level	FSH (IU/L)	.064	340	.340	.941	.830	1.066
	LH (IU/L)	.026	.055	.055	1.051	.999	1.106
	E2 (pg/ml)	.005	.535	.535	.997	.988	1.006
	P (ng/ml)	.555	.740	.740	.832	.280	2.469
Hormone level on hCG day	FSH (IU/L)	036	.308	.308	.964	.899	1.034
	LH (IU/L)	.009	.580	.580	.995	.977	1.013
	E2 (pg/ml)	.000	.270	.270	1.000	.999	1.000
	P (ng/ml)	.192	.118	.118	1.350	.927	1.968
Cycle length(days)		.027	.020	.181	1.028	.987	1.070
No. of Follicles >10 mm (n.)		-.014	.022	.521	.986	.944	1.030
No. of Follicles >14 mm (n.)		.145	.071	.042*	1.156	1.005	1.328
No. of Follicles >18 mm (n.)		-.066	.082	.424	.936	.797	1.100
Average E2 level (pg/ml)		.001	.001	.219	1.001	1.000	1.002
Endometrial thickness (mm)		.088	.034	.009*	1.093	1.022	1.168

Pregnancy outcomes; clinical pregnancy. Average E2 level = E2 level on hCG day/No. of follicles >14 mm. Se; Standard error, OR; Odds ratio, CI; Confident interval, *; significant difference.

### Attempts of IUI for PCOS women

**Figure 2:**
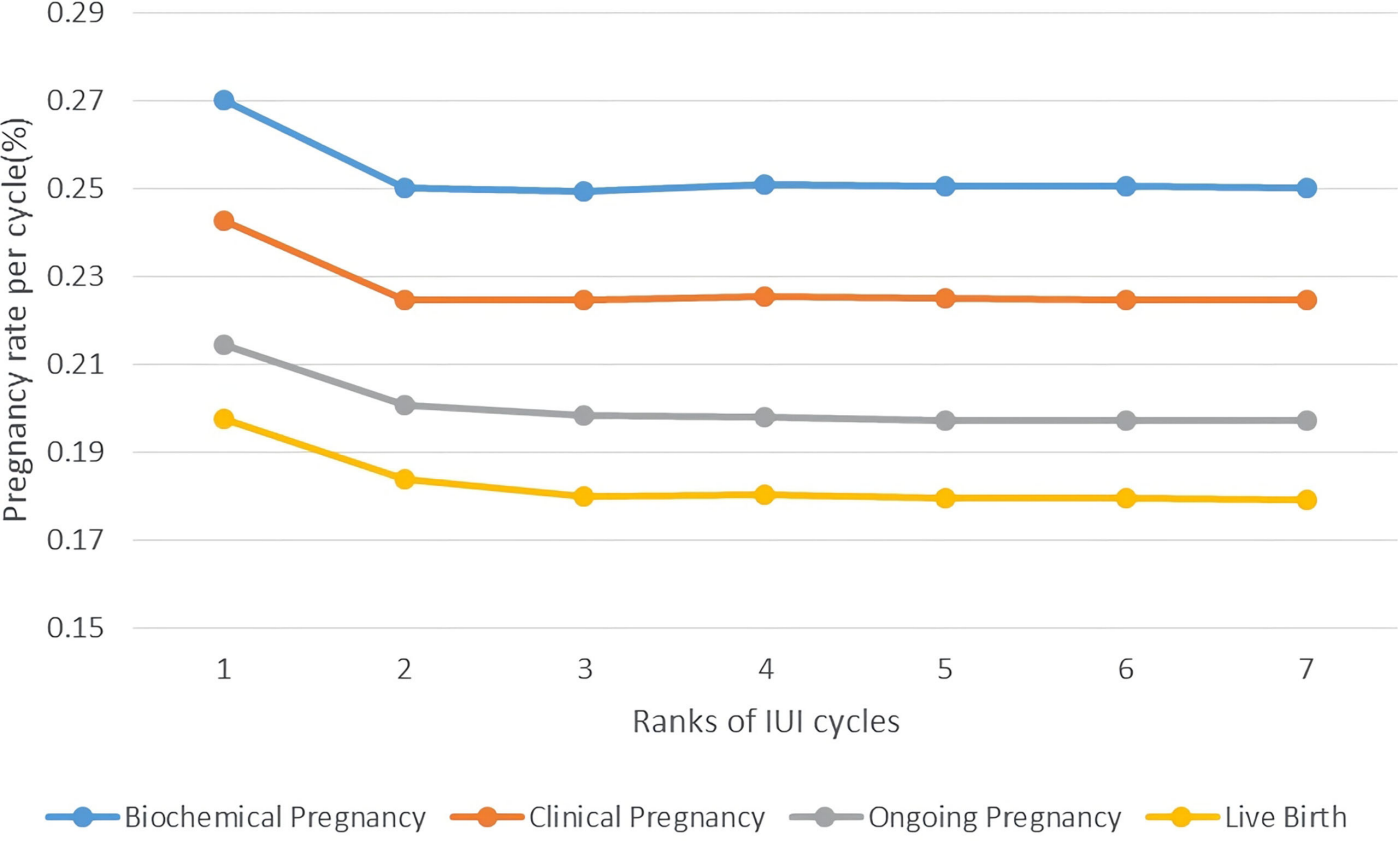
Pregnancy rate per cycle of PCOS patients undergoing different attempts of IUI in line charts. Pregnancy outcomes were as follows: biochemical pregnancy, clinical pregnancy, ongoing pregnancy, and live birth.

Then, we compared the pregnancy outcomes of different attempts of IUI, by making two line charts showing the pregnancy rate per cycle and cumulative pregnancy rate per patient. As is shown in **Figure 2**, the pregnancy rate per cycle was the highest in the first cycle, which hit 26.89% for biochemical pregnancy rate and 24.13% for clinical pregnancy rate. Pregnancy rates fell rapidly at the following 2-3 cycles and maintained around the level of 25% for biochemical pregnancy rate and 22.50% for clinical pregnancy rate after 3 cycles of IUI. The live birth rate of different ranks of IUI cycle followed the same pattern, namely 19.64% in the first cycle and keeping ~18% after 3 cycles of IUI.

**Figure 3:**
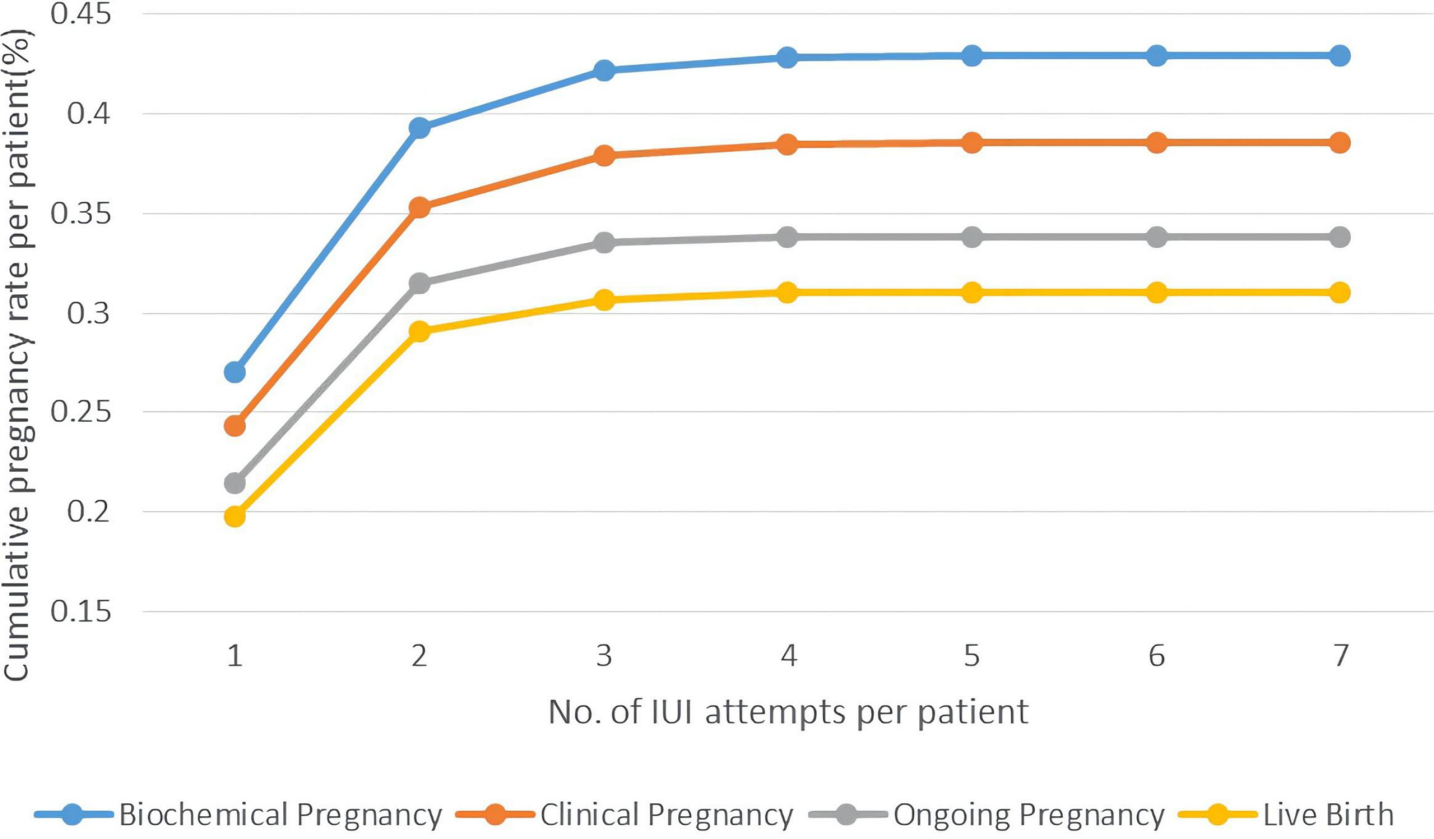
Cumulative pregnancy rate per patient of PCOS women undergoing different attempts of IUI in line charts. Pregnancy outcomes were as follows: biochemical pregnancy, clinical pregnancy, ongoing pregnancy, and live birth.

The change of cumulative pregnancy rate per patient followed a different pattern, which showed a rapid growth in the first two cycles and had a small rise in the 3rd-4th cycles. Starting from 26.89% for biochemical pregnancy rate and 24.13% for clinical pregnancy rate, 2 cycles of IUI had a cumulative biochemical pregnancy rate at 39.23%, clinical pregnancy rate at 35.17%. After 4 cycles of IUI attempts, the cumulative pregnancy rate per patient was relatively high in our study, 42.91% for biochemical pregnancy, 39.14% for clinical pregnancy, 33.70% for ongoing pregnancy, and 30.95% for live birth. (**Figure 3**)

### Outcomes of patients undergoing IVF/ICSI and those with 3 attempts of IUI

In order to compare the pregnancy outcomes of PCOS patients undergoing directly IVF/ICSI and those who underwent 3 times of IUI, we conducted a PSM as the baseline characteristics of two groups varied significantly. The following variables were extracted from our data: age, BMI, previously failed OI cycles, cycle length and basal hormone levels including FSH, LH, E2 and P. After PSM, baseline characteristics were well balanced between the 2 groups (**Table 5**). As shown in this table, the biochemical and clinical pregnancy rates of PCOS patients undergoing IVF/ICSI were 65.55% and 63.28%, higher than the cumulative pregnancy rates of patients who underwent 3 attempts of IUI (42.91% for biochemical pregnancy and 38.49% for clinical pregnancy rate).

**Table 5:** The basal characteristics of PCOS patients undergoing IVF and three attempts of IUI after PSM, and the pregnancy outcomes of these two groups of patients.

Variables		IVF Group (n=3222)	IUI Group (n=1086)	SMD
Age (Years.)		32.97 (31)	33.31 (31)	0.06
Cycle Length (Days)		12.37 (10)	12.53 (10)	0.07
Basal hormone level	FSH (IU/L)	5.44 (4.40)	5.24 (4.42)	0.03
	LH (IU/L)	5.27 (2.77)	5.28 (3.15)	0.06
	E2 (pg/ml)	34.92 (33.00)	33.98 (33.00)	0.04
	P (ng/ml)	0.27 (0.20)	0.25 (0.20)	<0.01
No. (%) of Biochemical Pregnancy		2112/3222 (65.55%)	466/1086 (42.91%)	NA
No. (%) of Clinical Pregnancy		2039/3222 (63.28%)	418/1086 (38.49%)	NA

SMD, standardized mean difference. In both IVF Group and IUI Group stands for number of patients included. Biochemical and clinical pregnancy rates in IVF group are pregnancy rates after a IVF cycle including ovum pick up and embryo transplantation, while biochemical and clinical pregnancy rates in IUI group are cumulative pregnancy rates after three attempts of IUI per patient. NA, Not Available.

### IUI outcomes of PCOS patients with more than 3 attempts of failed OI therapy

**Figure 4:**
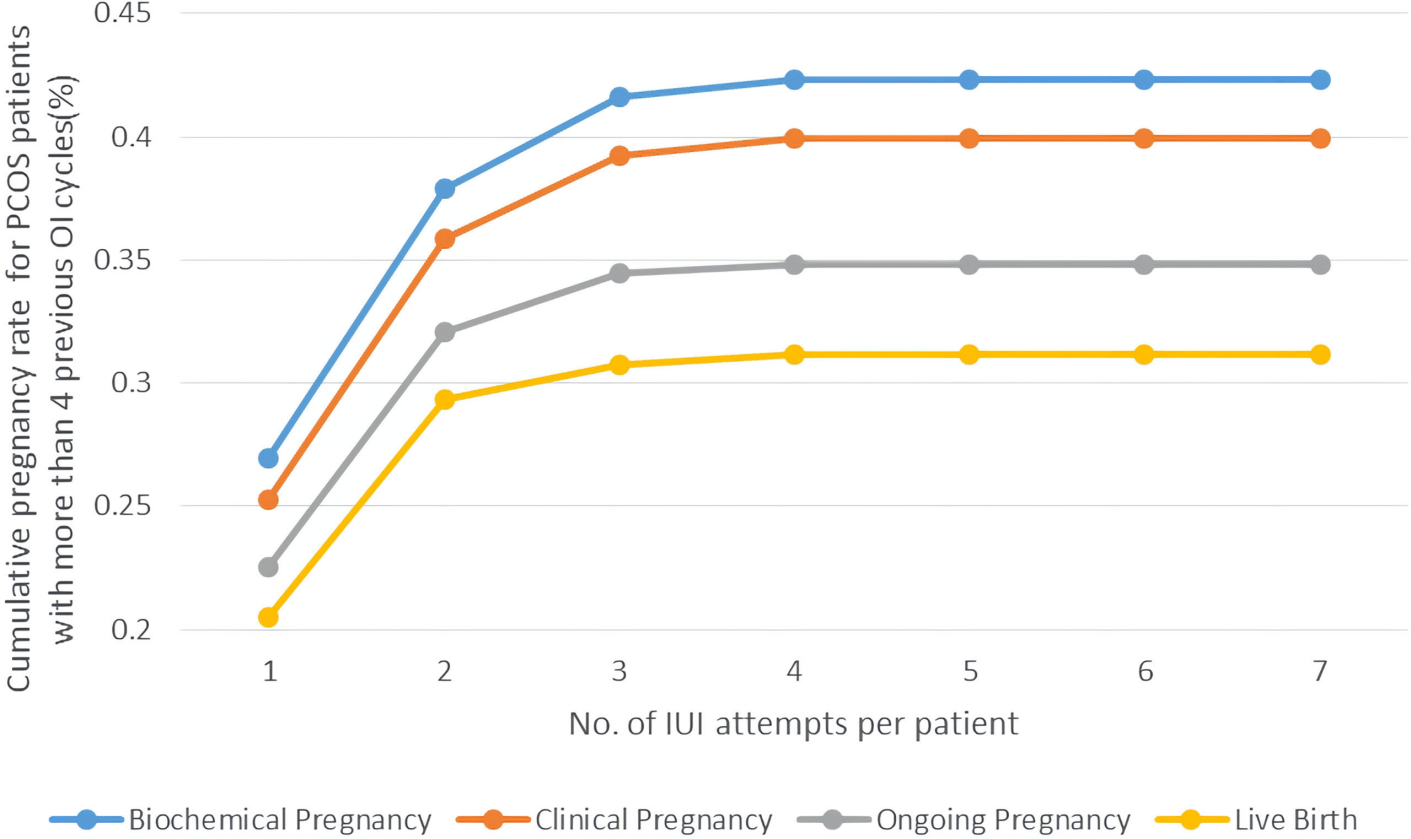
Cumulative pregnancy rate of PCOS women with >3 previous OI cycles undergoing different attempts of IUI in line charts. Pregnancy outcomes were as follows: biochemical pregnancy, clinical pregnancy, ongoing pregnancy, and live birth.

Some experts considered approximately 75% of pregnancies had occurred in the first three cycles of the OI treatment (3). We investigated the pregnancy rates for PCOS patients with more than 3 attempts of OI treatment before they switched to IUI to explore whether IUI could be a second-line therapy. As is shown in **Figure 4**, PCOS patients with more than 3 failed attempts of OI therapy had a similar level of pregnancy rate with all PCOS patients undergoing IUI as well as a similar trend of platform effect at three cycles of IUI. The Cumulative biochemical pregnancy/clinical pregnancy/live birth rate per patient was 26.96%/25.26%/20.51% in the first cycle, which rose to 37.88%/35.84%/29/30% in the second cycle and stabilized at ~42%/39%/31% after the first three cycles.

We further performed logistic regression to find out the predictive factors that affect pregnancy outcomes of IUI for PCOS patients with more than 3 failed OI cycles. The results manifested that the causes of infertility did not affect the clinical pregnancy rate with the P-values at 0.747, 0.463, and 0.866, while the No. of follicles >14mm on hCG day had a significantly positive connection with clinical pregnancy rate with a P-value at 0.08 (OR=1.812). Another factor that positively correlated with clinical pregnancy rate was the average E2 level per follicle with a P-value at 0.032 (OR=1.003). (**Table 6**).

**Table 6:** Logistic regression analysis for pregnancy outcomes of PCOS patients undergoing IUI with >3 previous OI cycles.

		B	Se	P	OR	CI	
						Lower	Upper
Infertility cause (n.)						
PCOS			.747			
PCOS + Male Factors		-.245	.333	.463	.783	.407	1.505
PCOS+ Endometriosis		.147	.873	.866	1.159	.209	6.420
Age(years)		-.052	.042	.215	.949	.874	1.031
Duration of Infertility(years)		-.141	.073	.052	.868	.753	1.001
BMI(Kg/m2)		.039	.039	.325	1.039	.962	1.123
Sperm Motility (%)		-.012	.008	.152	.988	.972	1.004
Basal Hormone Level	FSH (IU/L)	-.063	.098	.520	.939	.775	1.318
	LH (IU/L)	.070	.045	.119	1.073	.982	1.172
	E2 (pg/ml)	-.007	.008	.398	.993	.979	1.009
	P (ng/ml)	1.066	1.020	.296	2.905	.393	21.456
Hormone Level on hCG Day	FSH (IU/L)	-.031	.064	.624	.969	.856	1.098
	LH (IU/L)	.023	.017	.167	1.024	.990	1.058
	E2 (pg/ml)	-.001	.001	.093	.999	.997	1.000
	P (ng/ml)	-.337	.386	.383	.714	.335	1.522
Cycle Length(days)		-.029	.040	.467	.971	.898	1.051
No. of >10mm follicles (n.)		-.039	.058	.503	.962	.859	1.077
No. of >14mm follicles (n.)		.595	.223	.008*	1.812	1.171	2.804
No. of >18mm follicles (n.)		-.094	.192	.625	.910	.625	1.327
Average E2 level (pg/ml)		.003	.001	.032*	1.003	1.000	1.005

Pregnancy outcomes; clinical pregnancy. Average E2 level = E2 level on hCG day/No. of follicles >14mm. Se, Standard error; OR, Odds ratio; CI, Confident interval; *; significant difference.

## Discussion

ART for PCOS patients has always been a topic. As WHO recommended, OI should be considered as the first-line therapy. And for those subfertile PCOS patients with repeated failed OI cycles, it is necessary to figure out whether they could benefit from IUI. However, there is limited studies available, for which we started our single-centered retrospective study. In our study, we included 1,868 IUI cycles of 1,086 couples in total. With such a large sample size, we were the first to propose that the attempts of OI previously should not be a predictive factor for IUI outcomes of PCOS patients. Hence, for PCOS women with repeated OI failures, IUI can still be a beneficial option, and it is not necessary for those patients to directly switch to IVF/ICSI before IUI was considered.

By comparing their basic and cycle characteristics, we learned that the BMI of patients with more previous OI cycles were smaller. It might be due to the weight loss treatment, while patients receiving OI previously. Also, patients with more previous OI cycles had a longer duration of infertility. The possible explanation was that it took more time for PCOS patients with more previous OI attempts before they switched to IUI, resulting in an obvious delay in the process of treatment and prolongation of infertility duration.

For our 1,086 IUI cycles, the average clinical pregnancy rate was 22.38% per cycle and 39.14% per patient (cumulative rate). The live birth rate of PCOS patients undergoing IUI was 17.80% per cycle and 30.95% per patient (cumulative rate). These results showed that PCOS patients undergoing IUI had a relatively high possibility of pregnancy. In 2019, another research team revealed that a significant difference was found in clinical pregnancy rates between the IUI group (48.2%) and the TIC group (11%), both following OI therapy in PCOS patients. This result was pretty comparable to ours. From these findings, we tended to believe that IUI, as a non-invasive ART, might contribute a lot to infertile women with PCOS (7).

Moreover, the pregnancy rates in IUI did not decrease with the increase in the patients’ failed OI attempts previously. PCOS women with more attempts of previous OI cycles had a slightly higher pregnancy rate but the difference was not significant (p=0.507), which indicated that: 1) Patients with different attempt of previous OI cycles could be treated with IUI as a substitution. 2) Previous failure of OI was not necessarily the indication for IVF/ICSI. Also, our data showed that PCOS patients with 1-2 previous failed OI cycles might be more likely to have an ectopic pregnancy. According to literature, one study pointed out that ectopic pregnancy rate might associated with the number of dominant follicles and multiple pregnancy (11), whereas another study mentioned that IUI with husband’s sperm tended to have a higher ectopic pregnancy rate than donor sperm (12). Although all the cycles included in our study used husband’s sperm and the other possible impact factors were not significantly different among groups, we still could not conclude from our results that whether the previous OI attempts contributed to the occurrence of ectopic pregnancy, considering that ectopic pregnancy happened in a tiny minority of IUI cases in our study (11 out of 1,868 cycles) and the result might be observed by chance.

With the above results, we concluded that IUI could be a treatment for PCOS patients as long as they did not have contraindications, such as severe male factors and bilateral oviduct obstructions, while the proper attempts of IUI before a couple switched to IVF/ICSI were still unknown. Our results showed that about 98% of pregnancies happened in the first 3 cycles of IUI, which meant that over four attempts of IUI might be considered as low profits. And a 2002 study also discovered such a platform effect of IUI in endometriosis and tubal factors patients. The average pregnancy rate per cycle was 9.7% during the first to forth CC-IUI cycles and decreased to 2.8% during the 5th to 6th cycles. There were no pregnancies after the sixth CC-IUI cycle in 57 attempts (13). Our results, together with this study, suggested that three to four attempts of IUI might be appropriate before IVF/ICSI.

As for the comparison of pregnancy outcomes of PCOS patients undergoing directly IVF/ICSI and those who underwent three attempts of IUI, our results demonstrated that the difference was significant, the clinical pregnancy rate of IVF group was 63.28% per patient, and cumulative clinical pregnancy rate after 3 IUI cycles was 38.49% per patient. Even so, this difference might be acceptable in clinical practice considering that IUI was a less invasive and less expensive therapy, which supported our suggestion that IUI could be an approach for PCOS patients before IVF/ICSI.

As a study previously showed that ~75% of pregnancy occurs in the first 3 cycles of OI therapy (3), we further investigated PCOS patients with more than three attempts of OI cycles undergoing IUI. The pregnancy outcomes of these patients were comparable to all PCOS patients, a finding indicative of the comparable pregnancy potentials of PCOS patients with more than three attempts of OI cycles and those without. It was a pity that we did not have the detailed information on all the patients’ previous OI cycles, including OI protocols and other cycle characteristics. However, as previous literature concluded, different OI drugs had little effects on pregnancy outcomes. For instance, a study involving a total of 1,068 IUI cycles from 735 PCOS revealed that CC, letrozole, and gonadotropins were equally effective and safe (8). Thus, our suggestion for infertile PCOS patients who suffered from repeated failures of OI, was to consider IUI before directly switching to IVF/ICSI.

We also performed a binary logistic analysis for PCOS patients undergoing IUI in our study, in which the result showed that the number of follicles >14 mm and the endometrial thickness were influencing factor for clinical pregnancy. In 2002, Houmard et al. revealed that patients might have higher pregnancy rate if the number of follicles > 14 mm was more than three (14). Similarly, another study in 1992 concluded that the number of follicles >12 mm was correlated with fecundity (15). Both of the results supported our findings that the number of follicles >14 mm had a positive correlation with clinical pregnancy in IUI. Moreover, some researchers discovered that the endometrial thickness on trigger day had a positively correlated with clinical pregnancy rate in IUI (16, 17), which was in accordance with ours.

There were still some weaknesses in our study. First of all, our data did not include information of patients who underwent TIC after OI in our department. The loss of these data led to the lack of comparison between PCOS patients undergoing TIC and IUI after OI. Secondly, ovarian stimulation protocols for patients’ previous OI cycles were not recorded in detail. However, Huang et al. claimed that different OI drugs, namely LE, CC, and hMG, did not have an impact on the pregnancy outcomes of IUI for PCOS patients (8). Therefore, although it was a defect that we could not get the exact data of OI history for each patient, it would not affect the results in general. Last but not the least, the cumulative pregnancy rate in our study might be lower than the reality. This might be because that we neglected some patients who quit IUI or asked for IVF/ICSI in the first 3-4 cycles based on their personal reasons or the preferences of their doctors in charge.

Our study demonstrated that IUI could be an assistance of infertile women with PCOS with a cumulative clinical pregnancy rate of 39.14% per patient and 22.38% per cycle. A history of repeated failures of OI treatments previously would not have a negative effect on pregnancy outcomes in IUI cycles and most pregnancies occurred in the first three cycles of IUI. Therefore, we suggested three attempts of IUI for PCOS women, who suffered from OI failures, before switching to IVF/ICSI. To summarize, IUI is a non-invasive but effective treatment for PCOS patients with previous OI histories, meanwhile a less painful and more cost-effective approach, compared with IVF/ICSI.

## Data availability statement

The raw data supporting the conclusions of this article will be made available by the authors, without undue reservation.

## Ethics statement

The studies involving human participants were reviewed and approved by the Ethics Committee (Institutional Review Board) of Shanghai Ninth People’s Hospital. Written informed consent for participation was not required for this study in accordance with the national legislation and the institutional requirements.

## Author contributions

YK and SJ supervived the entire study, including the procedures, conception, design and completion. YG was responsible for the collection of data. SJ, LC, YG and SZ were responsible for data analysis and interpretation. SZ gave crucial and instructional advice on statistical methods and study design. All athors drafted the article and approved the final manuscript.

## Funding

This research was supported by Clinical Research Program of 9th People's Hospital, Shanghai Jiao Tong University School of Medicine (JYLJ201915), the National Key Research and Development Program of China (2018YFC1003000) and the National Natural Science Foundation of China (81901478).

## Acknowledgments

We gratefully acknowledge all the staff of the department of assisted reproduction in Shanghai Ninth People’s Hospital for their support and cooperation.

## Conflict of interest

The authors declare that the research was conducted in the absence of any commercial or financial relationships that could be construed as a potential conflict of interest.

## Publisher’s note

All claims expressed in this article are solely those of the authors and do not necessarily represent those of their affiliated organizations, or those of the publisher, the editors and the reviewers. Any product that may be evaluated in this article, or claim that may be made by its manufacturer, is not guaranteed or endorsed by the publisher.
